# *In vivo *gene targeting of IL-3 into immature hematopoietic cells through CD117 receptor mediated antibody gene delivery

**DOI:** 10.1186/1479-0556-2-16

**Published:** 2004-10-27

**Authors:** Alain Chapel, Olivier Deas, Morad Bensidhoum, Sabine François, Moubarak Mouiseddine, Pascal Poncet, Antoine Dürrbach, Jocelyne Aigueperse, Patrick Gourmelon, Norbert C Gorin, François Hirsch, Dominique Thierry

**Affiliations:** 1 Institut de Radioprotection et de Sûreté Nucléaire, Département de Protection et de santé de l'Homme et de Dosimétrie, Section Autonome de Radiobiologie Appliquée à la Médecine, Fontenay aux roses, France; 2 Laboratoire de Thérapie Cellulaire et de Radioprotection Accidentelle, LTCRA, UPRES 1632, CHU Saint Antoine, Paris, France; 3 Inserm U542 and Paris XI University, Villejuif, France; 4 Institut Pasteur, Paris, France

## Abstract

**Background:**

Targeted gene transfection remains a crucial issue to permit the real development of genetic therapy. As such, *in vivo *targeted transfection of specific subsets of hematopoietic stem cells might help to sustain hematopoietic recovery from bone marrow aplasia by providing local production of growth factors.

**Methods:**

Balb/C mice were injected intravenously, with an anti-mouse c-kit (CD117) monoclonal antibody chemically coupled to a human IL-3 gene-containing plasmid DNA. Mice were sacrificed for tissue analyses at various days after injection of the conjugates.

**Results:**

By ELISA, the production of human IL-3 was evidenced in the sera of animals 5 days after treatment. Cytofluorometric analysis after *in vivo *transfection of a reporter gene eGFP demonstrated transfection of CD117+/Sca1+ hematopoietic immature cells. By PCR analysis of genomic DNA and RNA using primer specific pIL3 sequences, presence and expression of the human IL-3-transgene were detected in the bone marrow up to 10 days in transfected mice but not in control animals.

**Conclusions:**

These data clearly indicate that antibody-mediated endocytosis gene transfer allows the expression of the IL-3 transgene into hematopoietic immature cells, *in vivo*. While availability of marketed recombinant growth factors is restricted, this targeting strategy should permit delivery of therapeutic genes to tissues of interest through systemic delivery. In particular, the ability to specifically target growth factor expression into repopulating hematopoietic stem cells may create new opportunities for the treatment of primary or radiation-induced marrow failures.

## Introduction

*In vivo *gene targeting of highly specific cell subsets remains the main challenge for gene therapy of a broad range of conditions associated with acquired diseases, including infectious disorders, cancer and failure of the hematopoietic system [1,2]. *In vivo *gene transfection is more appealing than *in vitro *transfection of an aliquot of cells or tissue that would be then reinfused to the patients, because it potentially concerns the total population of targeted cells disseminated in the whole body; this is particularly relevant to patients with primary or secondary failures of the hematopoietic system, since, in most instances, residual foci of hematopoiesis exist that cannot be easily located and cannot be collected by a marrow harvest procedure. *In vivo *targeted transfection of specific subsets of hematopoietic stem cells (HSC) might help to sustain hematopoietic recovery from bone marrow aplasia by providing local production of growth factors.

Systemic gene delivery systems are needed for therapeutic applications in which the target cells are not directly accessible [3]. However, for several reasons including lack of cell specificity and safety, *in vivo *targeted gene transfer cannot use current viral vectors. Although cationic liposomes have been promising systems in transfecting cells in tissue culture, it has been recognised that their *in vitro *efficiency does not correlate with their ability to deliver DNA after *in vivo *administration [4-10].

Tissue-specific targeting can be achieved through ligand receptor interactions [11,12]. We have already described a technique of antibody-mediated targeted gene transfection termed antibody delivery system [11,12]: a ligand (capable of binding to the surface of the targeted cells) conjugated with plasmid DNA retains its ability to specifically interact with cognate receptors on the cell surface.

In previous studies, antibodies directed against internalised cell surface antigens such as the T lymphocyte-related CD3 molecule or the B lymphocyte-related surface IgD were chemically coupled to purified plasmid DNA encoding various reporter genes. This approach was validated both *in vitro *by the transfer of G418 resistance (neo^r^) into human T-cell lines [13] or human hematopoietic immature cells [14] and *in vivo *by the transfer of β-galactosidase activity into mouse splenocytes [13]. We have reported that this strategy can be applied to targeted gene delivery to human renal carcinoma cells [15]. More recently, *in vivo*, we have shown a specific tumor targeting after a single intravenous injection in mice bearing tumour expressing the renal carcinoma – related G250 tumor associated antigen [16].

We have previously reported that the method is suitable for the production of a functional growth factor in specifically CD117+ targeted cells, mediating an *in vitro *biological effect on hematopoiesis [14]. As our previous report evidenced interaction of the conjugate with hematopoietic cells *in vitro*, this study was focused on specific *in vivo *targeting of hematopoietic tissues.

In the present study, we used anti-CD117 (c-kit) mAb covalently coupled to human *IL-3*-encoding plasmid DNA. CD117 antigen is expressed on a CD34+ hematopoietic subpopulation and is readily internalised upon binding to its ligand [17]. Thus, targeted-gene transfer through CD117 may be achieved in this cell subset. We indeed demonstrated an *in vivo *targeting of hematopoietic immature cells via a systemic route, mediating an efficient *in vivo *transgene expression.

## Methods

### Ab-DNA conjugation

The human IL-3 coding sequence (R&D Systems, Minneapolis, Minnesota) was ligated to synthetic fragments containing the natural leader sequence of human IL-3 and was subcloned into pCEP4 vector (Invitrogen Corporation). Transgene expression was controlled by the cytomegalovirus (CMV) enhancer-promoter sequence. The Epstein-Barr Virus replication (oriP) and nuclear antigen (encoded by the EBNA-1 gene) were carried by this plasmid to permit extrachromosomal replication in human, primate and canine cells [18]. pCEP4 also carries the hygromycin B resistance gene for stable selection of transfected cells. The resulting vector was named pIL3.

IgG mAbs were chemically coupled to plasmid DNA as previously described [13]. Briefly, purified IgG (3 mg/ml) in borate buffer (pH 8.2) (100 mM boric acid, 25 mM sodium tetraborate, and 75 mM NaCl) were activated using 3 mg/ml (final concentration) of benzoquinone (Sigma-Aldrich, St Louis, Missouri, USA). After gel filtration through a G25 column (Roche Diagnostics, Mannheim Germany) activated IgG were then covalently linked to pIL3 24 hours, in 0.1 M carbonate buffer (pH 8.7), in a ratio of 100 μg of plasmid DNA for 10 μg of IgG antibody. IgG-plasmid conjugates were then purified by HPLC. Antibodies used was clone 2B8 a monoclonal rat anti mouse IgG reacting with the mouse p145 c-kit protein (CD117) (BD Biosciences Pharmingen Tullastrasse, Heidelberg, Germany). The negative control was the mouse G250 IgG1 mAb reacting with human renal cell carcinoma (kindly provided by Dr A. Gorter, The Netherlands) [19]. The quantities of conjugates were expressed as the quantities of plasmid initially used for reaction.

### In vivo transfection assessment

We have previously shown that *in vitro *transfection of HSC may be observed in a dose-dependent effect for up to 100 μg of conjugate [14].

BalbC mice (6 weeks) were intravenously injected with a dose of up to 400μg of monoclonal 2B8 (BD Biosciences Pharmingen) covalently coupled to the pIL3 plasmid (named conjugate) and as negative control the monoclonal 2B8 and plasmid DNA uncoupled (named unconjugate) or irrelevant human monoclonal antibody (G250) covalently coupled to the pIL3 plasmid (named control conjugate) or physiological serum (named control serum).

In a set of experiments, two intraperitoneal injections of chloroquine (32.5 mg/kg) were performed 2 hours and just a few minutes before intravenous injection of conjugates. The tolerance of chloroquine (used to prevent the degradation of the plasmid for transfection assays, 20) was in the range reported in mice for the study of malaria treatment [21]. Monoclonal antibody (mAb) 2B8 (BD Biosciences Pharmingen) was covalently coupled to 100 μg of the *enhanced green fluorescent protein *encoding plasmid pEGFP-1 provided from Clontech and was named eGFP conjugate.

Mice were intravenously injected twice (day 0 and day 2) and euthanasied 5, 7 or 10 days after the first injection of the conjugate, after proper anaesthesia.

Human IL-3 production in serum was assayed by High Sensitivity ELISA (R & D Systems). Controls were sera or cell culture supernatants of control mice (unconjugate, control conjugate, control serum).

After euthanasia, the presence of the transgene was investigated in blood, brain, lungs, liver, spleen, kidneys, adrenal glands and bone marrow. In order, to observe toxicity the weight of mice and their organs were measured (brain, lungs, liver, spleen, kidneys).

In mice injected with eGFP conjugate, a MACS magnetic cell separation systems (Miltenyi Biotec, Sunnyvale, CA) was used to enrich cells expressing CD117 and Sca1 from mononuclear bone marrow cells. Negative and positive cells were collected for experimental use. To achieve a purity greater than 50%, it was necessary to perform two sequential passes through magnetic columns. The overall recovery of CD117 was about 30% and enrichment 40 fold, as assessed by the fraction of CD117/Sca1 positive population before and after separation. Cells were analysed by flow cytometry to determine the purity of cell fractions. Then the presence of eGFP positive cells was investigated by flow cytometry into negative fraction (CD117/Sca1 negative populations) and positive cell fractions (CD117/Sca1 positive populations). All experiments were conducted according to French regulation for animal experimentation (Ministry of agriculture Act No.87848, 1987).

### Long-term cultures

Long-term cultures of bone marrow cells were performed, as previously described [22]. At one week, 50 μg/ml of hygromycin were added to the long-term culture, in order to select for stably transfected cells (plasmid conferred hygromycin resistance to stably transfected cells). After 1-week selection, these cells were cultured 2 weeks in long-term culture medium. Viable cells were numbered using trypan blue exclusion assay.

### Clonogenic hematopoietic progenitor assay

5 × 10^5 ^cells from bone marrow were assayed for clonogenic hematopoietic immature cells [23]. Briefly, cells were plated in triplicate in 35-mm dishes at a concentration of 5 × 10^5 ^cells/ml in complete methylcellulose M3434 from Stem Cell Technologies (West Broadway, Vancouver, Canada). Cultures were incubated at 37°C in 5% CO_2 _and removed at 14 days. Colonies were defined as containing more than 40 cells using an inverted microscope. Cells were then harvested and studied for *IL-3 gene *expression. Two weeks post-transfection, semi-solid colonies were removed from methylcellulose culture for PCR analysis of the presence of the pIL3 plasmid.

### DNA and RNA analyses

The simultaneous isolation of total cellular RNA and DNA from tissues or cells was performed using TriPure Isolation Reagent Kit (Roche Diagnostics) [24]. Total cellular RNA was incubated 30 min in the presence of RNAse-free DNAse (Invitrogen), heated at 90°C for 5 min and promptly cooled at 4°C. The RT-PCR was then carried out as previously described [25]. Briefly, total cellular RNA was first annealed with 1 mM of oligo-dT15 (Sigma-Aldrich) and then incubated at 42°C for 1 hour in the presence of 100 units of Moloney murine leukemia virus reverse transcriptase (Invitrogen) in a final volume of 20 μl. DNA or the reverse transcriptase reaction mixtures were then subjected to PCR amplification using sense primer (GTGGTTTGTCCAAACTCATC) and anti-sense primer (AGAGCTCGTTTAGTGAACCG) located on both sides of the IL-3 gene (into the multiple cloning site of pCEP4), which resulted in a PCR product specific of the gene inserted in the pCEP4. Nested PCR was performed using sense (CCAAACTCAATGTATCTTATCATGTCT) and anti-sense (TCAGATTCTAGAAGCTTGGGT) primers localized in the multiple site of clonage of pCEP4 plasmid. These pairs of primers allow for detection of a 542 bp fragment when electrophoresed on a 2% agarose gel and visualization with ethidium bromide. Specificity of PCR products was controlled using an internal ^33^P-5'-end labeled oligo-probe specific of *human IL3 coding *sequence (ACGGCCGCACCCACGCGACA), in Southern blot analysis as previously described [26]. To detect a false positive due to plasmid contamination, we have tested RNA samples by direct amplification of RNA (without the reverse transcription step). Indeed in the absence of plasmid, Taq pol will be unable to amplified RNA whereas a PCR product would be observed if the RNA sample was contaminated with plasmid DNA. No DNA plasmid contamination was observed for all the assayed RNA samples. As internal control a 590 bp region of the endogenous mouse RAP-SYN gene was also amplified using a second set of unique 30 bp primers (sense: AGGACTGGGTGGCTTCCAACTCCCAGACAC, anti-sense: AGCTTCTCATTGCTGCGCGCCAGGTTCAGG), which allows the detection of a 590 bp fragment [27].

## Results

### Assessment of transgene product secretion

Balb/C mice were intravenously injected twice (day 0 and day 3), with the anti-mouse CD117 (c-kit) 2B8 mAb conjugated to pIL3 expression vector. Control animals received unconjugated pIL3 expression vector and 2B8 mAb (named control unconjugate) or irrelevant G250 mouse mAb covalently coupled to the pIL3 plasmid (named control conjugate) or physiological serum (named control serum). To increase the transgene processing into cells, mice were injected with the conjugate up to a dose of 400μg in the presence or not of chloroquine known to diminish endosomal DNA degradation [20]. Mice were euthanasied 5, 7 or 10 days after the first injection of the conjugate. The presence of human IL-3 in serum was measured by a human IL3 specific ELISA, from 5 to 10 days. Using 400μg of conjugate in the presence of chloroquine, we detected human IL-3 in the serum of mice at 50 pg/ml at day 5 (table 1). No human IL-3 was observed in the serum of mice sacrificed at days 7 and 10 nor in mice injected with lower dose of conjugate, with control unconjugate or control conjugate (data not shown).

**Table 1:** Detection of circulating human IL-3 in mouse serum at day 5 post injection of pIL3 conjugate

Treatment (IP injection)	Quantity of conjugate	pg/ml of human IL-3 in mice			
Chloroquine		unconjugate		conjugate	
		mean	sd	mean	Sd
0	100μg	0	0	0	0
0	400μg	0	0	0	0
2 × 32.5 mg/kg	100μg	0	0	0	0
2 × 32.5 mg/kg	400μg	0	0	50*	17

The presence of human IL-3 in serum was investigated by ELISA. The data are representative of three independent experiments and are the mean of triplicate determinations ± S.D. * indicates statistically significant differences by Student's *t*-test analysis; p < 0.007 as compared to 400μg of unconjugate.

### Assessment of transfection cell specificity

Gene targeting was then evaluated by injecting mice with eGFP conjugated or unconjugated to either 2B8 mAb or to G250 control mAb. At day 5, the presence of transfected cells into bone marrow mononucleated cells was analysed into the purified CD117- and CD117+ subpopulations, by flow cytometry using anti-CD117 and anti-Sca1 Abs. As shown in Table 2, 4.7% cells from the CD117+/Sca1- and 2.8% cells from the CD117+/Sca1+ subpopulations collected from mice injected with the eGFP-2B8 conjugate were positive. All controls were negative.

**Table 2:** Detection of transfected cells in bone marrow mononucleated cells at 5 day postinjection of eGFP conjugate

plasmid	eGFP			
Cell population	Control serum	Unconjugate	Control conjugate	Conjugate

MNC	0	0	0	0
CD117-	0	0	0	0
CD117-/Sca1-	0	0	0	0
CD117+/Sca1-	0	0	0	4.7%
CD117+/Sca1+	0	0	0	2.8%

The presence of transfected cells (eGFP positives) in bone marrow was investigated 5 days postinjection among mononucleated cells (MNC): CD117 negative cell population (CD117-), CD117/Sca1 negative cell population (CD117-/Sca1-), CD117 positive/Sca1 negative (CD117+/Sca1-) and CD117/Sca1 positive cell population (CD117+/Sca1+). In all cases no transfected cells were observed in the controls.

### Assessment of transfection tissue specificity

**Figure 1:**
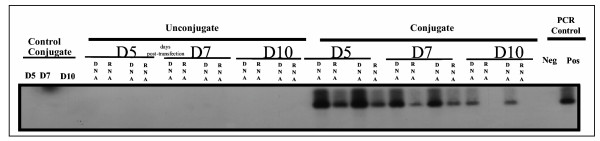
Nested PCR detection of pIL3 plasmid in bone marrow 5, 7, and 10 days after injection of the conjugate. Mice were intravenously injected twice with 100μg of anti-CD117-pIL3 conjugate (at day 0 and at day 2). Control groups corresponded to bone marrow of mice treated with unconjugated pIL3 and anti-CD117 Abs or control conjugate (G250-pIL3). IL3 DNA and RNA were detected in the bone marrow of animals receiving the pIL3-anti CD117 conjugate up to day 10. The data are representative of three independent experiments.

**Figure 2:**
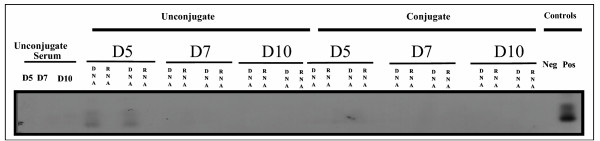
Nested PCR detection of pIL3 plasmid in mononuclear peripheral blood cells 5, 7, and 10 days after injection of the conjugate. Mice were intravenously injected twice with 100μg of anti-CD117-pIL3 conjugate (at day 0 and at day 2). Control group corresponded to mononuclear peripheral blood cells or serum of mice treated with unconjugated pIL3 and anti-CD117 Abs. pIL3 DNA was only detected in peripheral blood of control animals until day 5 after the first injection. The data are representative of three independent experiments.

To assess the tissue specificity of the targeting, presence of pIL3 plasmid was investigated in bone marrow, blood cells, liver, spleen, lungs, kidneys, adrenal glands, and brain. PCR analysis of genomic DNA and RNA isolated from bone marrow and blood (or serum) was performed using primer specific pIL3 sequences. Specificity of the PCR and RT-PCR products was assessed by a Southern blot hybridised with a specific radiolabelled human IL3 probe. The expected 542 bp band of the PCR product corresponding to the IL3-transgene presence (both DNA and RNA) were was specifically detected in the bone marrow of transfected mice up to 7 days for RNA and 10 days for DNA, post transfection (figure 1). Nested PCR also was positive for the *IL3 *transgene DNA in the spleen of transfected animals up to day 7 (not shown). In control animals (control serum, unconjugate, control conjugate), pIL3 DNA but no RNA was detected in peripheral blood but not in serum until day 5 after the first injection and then disappeared (figure 2); there was no detection of DNA or RNA in bone marrow (figure 1). Aside from this, all other tissues were negative when assayed by nested PCR on day 5, 7, 10 in transfected animals. *IL3 *transgene DNA was only found in the kidney of control animals receiving an unconjugated mixture of Ab and DNA or the control conjugate, on day 5 only (not shown).

The measurement of the weight of the mice and their organs (liver, kidneys spleen, brain, adrenal glands, lungs), did not reveal any change, suggesting the lack of toxicity detected in mice receiving the conjugate (data not shown). Furthermore, since no IL3 transgene was evidenced in these organs, further investigation of potential toxicity of conjugate might not be relevant.

Finally, clonogenic assay hematopoietic immature cells were performed on cells removed from sacrificed animals. As shown in Table 3, their was no differences in mice receiving the conjugate, control unconjugate, control conjugate and mice receiving physiological control serum. These data clearly demonstrated that our approach did not alter the hematopoiesis.

**Table 3:** Frequencies of colonies in bone marrow following transfection anti-CD117-pIL3 conjugate

Days	Control serum		Unconjugate		Control conjugate		Conjugate	
	mean	sd	mean	sd	mean	sd	mean	sd

5	191	10	183	8	192	10	185	25
7	157	55	152	50	192	12	197	16
10	187	23	173	11	182	22	187	26

Number of colonies was measured 5, 7 and 10 days following *in vivo *transfection with 100μg of anti-CD117-pIL3 conjugate. Control groups corresponded to mice injected with unconjugated pIL3 and anti-CD117 mAb or with the control conjugate (G250-pIL3). 5 × 10^5 ^cells from bone marrow were cultured in complete methylcellulose. Colony (aggregates of more than 40 cells) numbers were evaluated under inverted light microscope. The data are representative of three independent experiments and are the mean of triplicate determinations ± S.D.

### Lack of transgene integration

**Figure 3:**
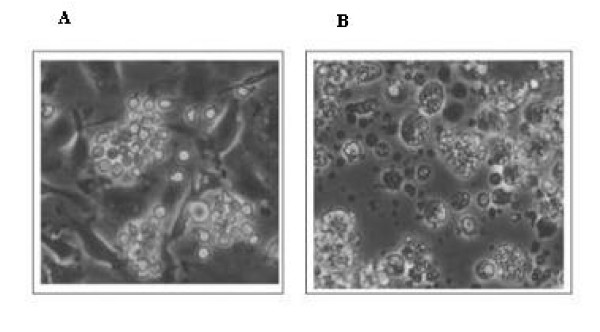
Morphology of survival long-term bone marrow cells. (a) Long-term bone marrow cells were cultured 7 days. (b) After a 1-week culture, 50μg/ml of hygromycin was added in order to select for stably transfected cells. After 1 week of selection, these cells were cultured 2 weeks in long-term culture medium. Cells observed in controls or in long-term culture in mice injected with the conjugate were viable (original magnification ×400).

Long-term cultures of bone marrow cells from mice receiving the conjugate or the controls were performed. After 1 week of selection in hygromycin-containing medium (plasmid conferred hygromycin resistance), cells were cultured for another 2 weeks and then viable cells were quantified using trypan blue exclusion assay. As illustrated on Figure 3, upon hygromycin selection, no viable cell was found in mice transfected with anti-CD117-pIL3 conjugate, suggesting that there was no integration of pIL3 into host DNA.

## Discussion

Although much progress has been accomplished in the field of gene therapy over the last years, there is still a need to develop more effective vectors and new strategies [28]. Using a non-viral gene delivery system, targeting primary hematopoietic stem/progenitor cells in *vitro *can be especially useful for studying the biological effects of various growth factors [29]. Our conjugate linking an anti-CD117 mAb to a pIL3 plasmid should be a good candidate to target specifically hematopoietic stem cells. We have previously reported that the method is suitable for the production of a functional growth factor in specifically CD117+ targeted cells, mediating an *in vitro *biological effect on hematopoiesis [14]. Since our previous report evidenced interaction of the conjugate with hematopoietic cells *in vitro*, the present study focus on specific targeting of hematopoietic tissues, *in vivo*.

We first demonstrated the efficacy of our approach since the transgene and its product (RNA and circulating human IL3) were found in mice injected with anti-CD117/pIL3 conjugate. It is of note that although human IL3 was only detected in plasma of chloroquine-treated mice injected with high quantity of conjugate (400μg); human IL3 encoding RNA were evidenced in treated mice injected with lower quantity of conjugate (100μg). These results were in accordance with the design of these experiments aiming at observing even a transitory and local effect (within the bone marrow).

PCR analyses of tissues evidenced the specific targeting of the hematopoietic system since brain, liver and lungs were negative. Only the spleen of mice transfected with the conjugate and kidneys of control animals (transfected with unconjugate mixture of Ab and DNA or with the control conjugate) displayed a positive PCR signal. Observed shortly after the last plasmid injection in blood, the presence of plasmid might be due to the intravenous administration route used and in kidney, to a progressive elimination of the plasmid in this organ of refinement. These results correspond to kinetic of plasmid availability when not using the specific vector (conjugate) to carry plasmid into progenitor cells. In the latter case, CD117+ cells were specifically transfected, and among them, Sca1+ cells were positive, suggesting a targeting of hematopietic progenitor cells via the systemic route.

Several parameters contribute to the efficiency and specificity of our system such as the internalisation of the antigen targeted, the choice of the transgene used, the tissues targeted, the conformation of the conjugate. Bone marrow was a good candidate for gene targeting as it is a highly proliferative tissue, as opposed to tissues which possess terminally differentiated cells such as hepatocytes or adipocytes, which are more resistant to transfection [30].

Factors affecting the bioavailabilty of the administered conjugates strongly determine their *in vivo *performance. These include avid interaction with serum components, resulting in colloidal instability, including both aggregation and dissociation of the conjugates and rapid elimination from blood circulation [31,32]. Therefore, the gene delivery carrier should function as a protector of DNA during *in vivo *administration. Protamine has been shown to cause condensation of DNA, which promotes cellular entry [33,34]. Our complex of plasmid and antibody may have been sufficiently compacted to resist nuclease degradation and non-specific interaction with plasma proteins. Furthermore the reduced dimensions of the conjugate may have been sufficient to allow its diffusibility through the extracellular space to reach bone marrow cells.

## Conclusions

Our *gene delivery system *is specific and leads to transient gene delivery and expression. It may prove useful and safe for numerous clinical applications of gene transfer in hemato-oncology and radiopathology, whereby a stable genetic modification is not required, in contrast to the gene therapy approaches for genetic diseases. For example, it may be of interest to facilitate the long-term reconstitution of hematopoiesis through transient gene delivery into progenitor cells of patients after therapeutic and /or accidental exposure to chemo/radiotherapy. Whether our approach could be used to potentate hematopoietic reconstitution following irradiation remains to be studied.

## List of Non-Standard Abbreviations Used

HSC Hematopoietic Stem Cells

## Competing Interests

The author(s) declare that they have no competing interests.

## Authors' contributions

AC, OD, AD, MB, SF, MM, PP carried out the studies. FH, DT participated to the designed of the study and its coordination. All authors read and approved the final manuscript.
